# Photo-induced drug release at interfaces with arylazopyrazoles†

**DOI:** 10.1039/d4sc04837g

**Published:** 2024-10-17

**Authors:** Ipsita Pani, Michael Hardt, Dana Glikman, Björn Braunschweig

**Affiliations:** a Institute of Physical Chemistry, Center for Soft Nanoscience (SoN), University of Münster Corrensstraße 28-30 Münster 48149 Germany braunschweig@uni-muenster.de

## Abstract

Smart responsive materials have spurred the progress in high-precision drug delivery. Enormous attention has been given to characterizing drug release in bulk aqueous solutions, however, aqueous–hydrophobic interfaces are vital components of biological systems which serve as the point of entry into cells. These interfaces are involved in many key biomolecular interactions, and while the potential for drug molecules to adsorb to these interfaces is recognized, their specific role in the context of drug release remains largely unexplored. We present a fundamental investigation on the release of encapsulated drugs at the air–water interface as a representative model to mimic the organic/aqueous interface of cells. Combining the advantages of light as an external stimulus and the superiority of arylazopyrazoles (AAP) over conventional azobenzene photoswitches, we report a micellar nanocarrier for the capture and release of the chemotherapeutic drug doxorubicin. Using a powerful combination of interface-sensitive techniques such as the Langmuir–Blodgett technique, surface tensiometry, and the interface-specific vibrational sum-frequency generation spectroscopy, we demonstrate the photoresponsive release of doxorubicin encapsulated in the micelles of AAP photosurfactants to the air–water interface. Complementary fluorescence measurements corroborate additional drug release in bulk aqueous solutions.

## Abstract 2

Using interface-sensitive techniques, we investigated the photoresponsive release of the chemotherapeutic drug doxorubicin from micellar nanocarriers of arylazopyrazole photosurfactants at the air–water interface as well as bulk aqueous solution.

## Graphical abstract

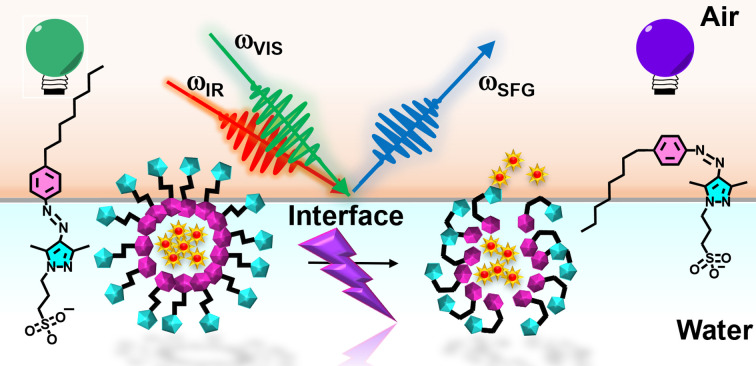

## Introduction

Stimuli-responsive materials have found an assortment of applications in high precision drug delivery.^1–4^ Continuous efforts have been dedicated to the development of ‘smart’ drug delivery systems to achieve controlled release and reduce non-specific toxicity.^5,6^ Most studies on the design of stimuli-responsive drug nanocarriers have focused on the release of an entrapped drug in bulk aqueous solutions.^3,4,7^ However, in biological systems, the majority of the water molecules are structured around hydrophobic surfaces of biomolecules.^8^ The “biological water” is only a few monolayers thick and has characteristics different from the bulk water, which are manifested in biomolecular functions.^8^ Therefore, aqueous–hydrophobic interfaces are vital in biology and medicine.^9^ The quest to find ideal nanocarriers for tailored drug release applications requires an understanding of the fundamental self-assembly of the carrier and drug molecules at aqueous–hydrophobic interfaces. Air–water interface has been used as a reliable model to recapitulate various interfacial phenomena occurring at the aqueous–organic interfaces in living cells.^10^ The insights on the molecular interactions between the drug and nanocarrier at interfaces are important for high precision drug delivery. In addition to that, understanding the precise interfacial contribution to drug release is challenging because the molecular information from the interface is not accessible to conventional analytical techniques used to characterize drug release. Our study is focused on elucidating the light-induced release of a drug at the air–water interface.

Light-responsive nanomaterials have found tremendous applications in drug delivery owing to the site-specific administration, tunability of dosage, minimum interference with healthy tissues, and spatiotemporal control.^11,12^ Photoisomerization is one of the simplest mechanisms of drug release, where the release is facilitated by *E*/*Z* photoisomerization causing a change in the polarity and hydrophilicity of the photoswitchable amphiphile.^12^ The majority of reports on photoisomerization-induced drug release are dominated by azobenzene and spiropyran amphiphiles.^11^ However, the spiropyran amphiphiles as well as the *Z*-isomer of most azobenzene derivatives have low thermal stabilities.^13^

Arylazopyrazoles (AAPs) have recently emerged as more promising molecular photoswitches with longer half-lives of the *Z*-isomer (up to ∼1000 days), near-complete *E*/*Z* photoswitching, and large changes in dipole moment upon photoisomerization.^6,14,15^ Owing to the superior photophysical properties, AAPs have gained much attention for interesting applications in long-term energy storage, light-responsive host–guest complexes, shape-memory materials, *etc.*^6,16–18^ Photoisomerization-induced changes in the self-assembly of AAP-based photoswitches in solution have been investigated thoroughly. Comprehensive studies on AAPs have demonstrated that alkyl AAPs form better light-reversible host–guest complexes than conventional azobenzenes.^15,19^ Small-angle neutron and X-ray scattering studies of an amphiphilic AAP surfactant by Tyagi *et al.* revealed an oblate to sphere transition in the micellar structure upon *E*–*Z* photoisomerization.^18^ Light-induced modulation of stiffness of AAP-based hydrogels has been utilized for controlled drug release.^17^

**Fig. 1:**
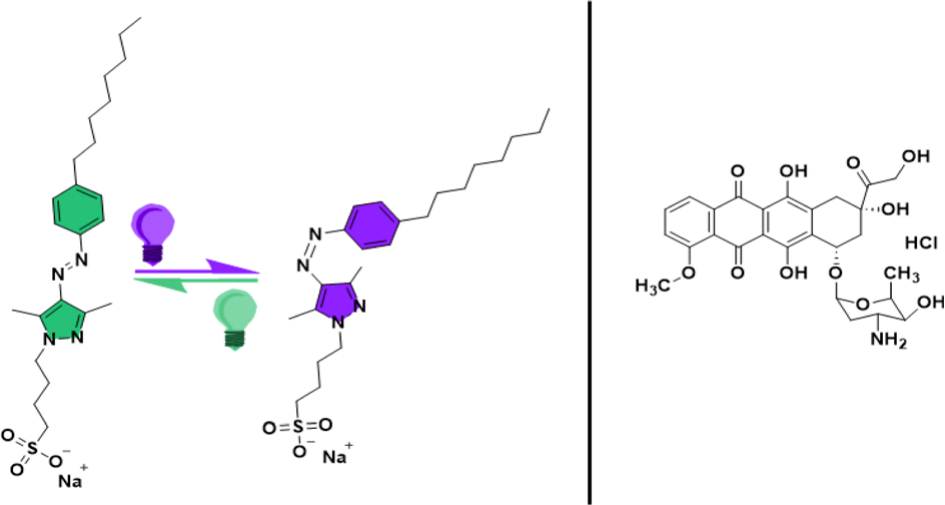
Chemical structures of the *E* (coloured in green) and *Z* (coloured in violet) configurations of the AAP surfactant (octyl-arylazopyrazole butyl sulfonate) and the doxorubicin hydrochloride (Dox) drug used in this study.

Many investigations have also been carried out to understand the light-induced structural changes in AAP amphiphiles with systematic structural modifications at air–water interfaces.^20–23^ A comparison of the interfacial properties of AAP and azobenzene amphiphiles at the air–water interface revealed more pronounced light-induced changes in the molecular order of the AAP surfactant.^20^ Large changes in surface tension (10–27 mN m^−1^) of alkyl AAP surfactants upon photoisomerization suggest drastic light-induced changes in surface activity.^20,22,23^ An observation of utmost relevance to the present study is that, for AAP surfactants, the critical micellar concentration (CMC) of the *Z*-isomer is significantly higher than the CMC of the *E*-isomer. In contrast to this, the CMC of a similar azo amphiphile was independent of the photoisomerization.^20^ In this work, we have used octyl-arylazopyrazole butyl sulfonate (AAP) as a photoswitchable surfactant to construct photoresponsive micellar nanocarriers (see structure in Fig. 1). The synthesis and detailed characterization of this AAP surfactant has been reported in a recent work by Hardt *et al.* where it has been shown that the *E* → *Z* photoisomerization shifted the CMC of AAP from 0.1 mM to 1 mM.^23^ Dramatic changes in the surface activity upon *E*/*Z* photoisomerization, high photostationary states, and remarkable thermal stability of the *Z*-isomer make this AAP surfactant a suitable choice for our micellar nanocarrier. We have used the AAP surfactant as a single component micellar nanocarrier for the capture and release of the most potent chemotherapeutic agent *i.e.* doxorubicin (Dox).^24^

In majority of the previous studies, often the intensity and duration of the UV irradiation is not mentioned, despite being highly important parameters for potential clinical applications. Studies that mention these parameters have applied high UV irradiance (*e.g.* 1 W cm^−2^) and periodic exposure times were necessary to achieve near-complete photoinduced release of Dox within tumour cells.^2,25^ In contrast, our work, which utilizes highly efficient AAP photoswitches, demonstrated that much lower intensities of the UV radiation (≤10 mW cm^−2^) as well as short exposure times (*t* ∼ 35 s in bulk and *t* ∼ 12 min at interface) are sufficient to trigger the release of Dox to the air–water interface. This significantly advances our approach toward realistic applications requiring UV radiation below the maximum permissible UV exposure for human skin (8.3 mW cm^−2^ for 2 min).^26^ While UV wavelengths pose challenges for light delivery systems due to their limited tissue penetration, lower light intensities can avoid radiation damage. As highlighted by Pearson *et al.*,^26^ alternative methods have been explored to address these challenges. Despite these limitations, UV phototherapy is a preferred routine treatment in dermatology.^27^ In our work, we focus on Dox encapsulated within *E*-AAP micelles, which are efficiently released to the air–water interface upon brief, low-intensity UV irradiation *via* the dissolution of *Z*-AAP micelles. To investigate this release process, we employed a powerful combination of surface-sensitive techniques.

## Results and discussions

To understand the effect of *E*/*Z* photoisomerization on the packing of AAP surfactants and Dox at the air–water interface, we recorded the compression isotherms of the *E*- and *Z*-AAP spread over a Langmuir trough containing aqueous solution of Dox at varying concentrations. Fig. S2 and S3† show plots of the surface pressure isotherms of *E*- and *Z*-AAP with and without Dox in the aqueous subphase which are discussed in detail in the ESI.† A comparison of the surface pressure isotherms of *E*- and *Z*-AAP without Dox suggests a higher surface activity of the *E*-AAP. With Dox in the aqueous subphase, we observe an overall expansion of the monolayers suggesting incorporation of Dox in the AAP monolayer. However, at 8 μM Dox, we see a flattening of the compression isotherm suggesting a partial desorption of molecules from the air–water interface to the aqueous subphase. To avoid this, we fixed the concentration of Dox to 5 μM.

**Fig. 2:**
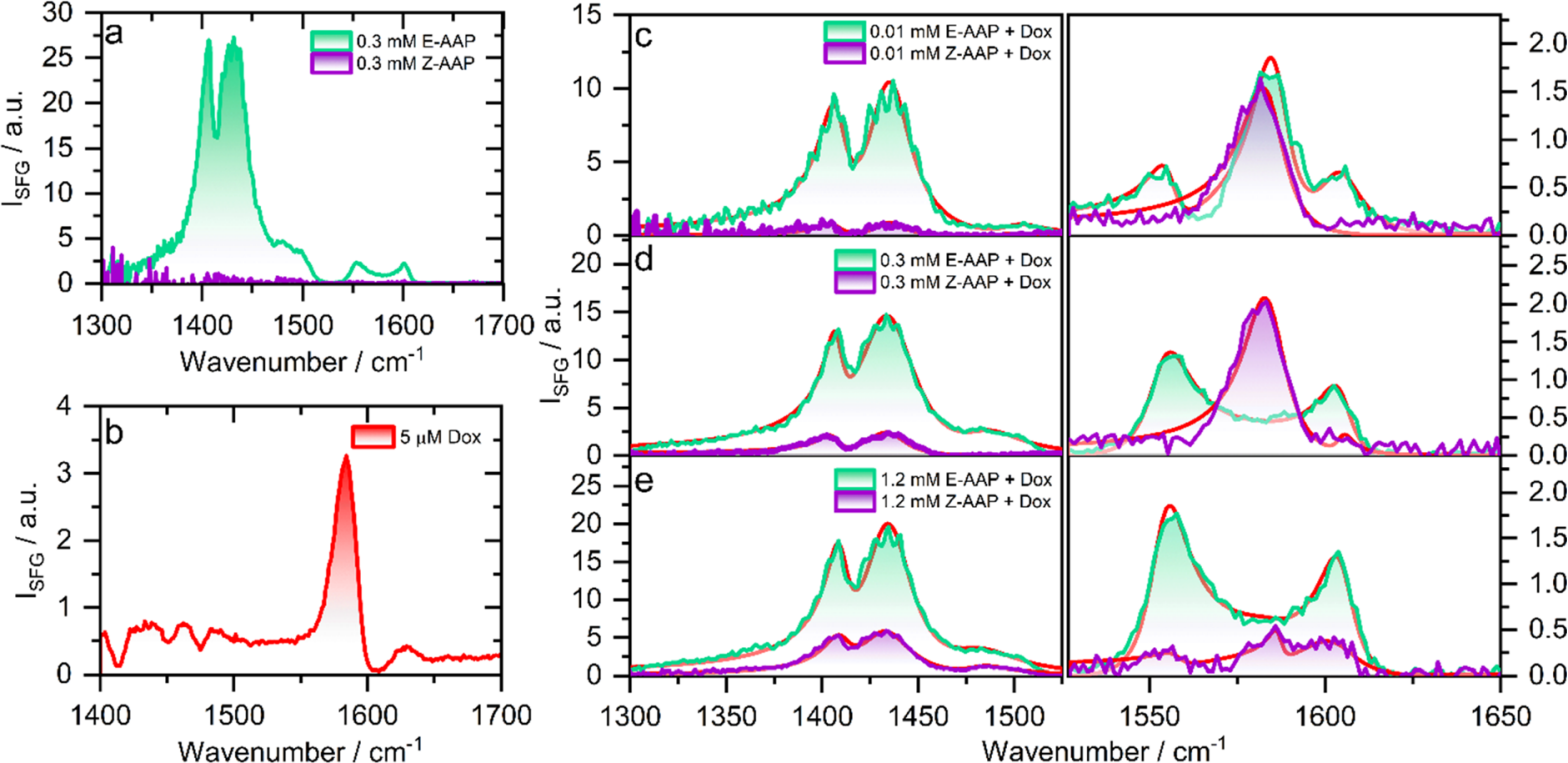
Vibrational SFG spectra of air–water interfaces modified by (a) 0.3 mM AAP, (b) 5 μM Dox, and (c–e) AAP–Dox mixtures under green (*E* isomer) or UV light (*Z* isomer) as indicated by the colour of the spectrum. In (c and d), the concentration of Dox was kept at 5 μM while the AAP concentration was varied from 0.01 to 1.2 mM as indicated. The SFG spectra of the UV-irradiated samples were recorded after 30 min of UV irradiation. Fits to the spectra are shown as red solid lines in (c and d).

To probe the changes in the molecular structure of Dox–AAP mixtures upon *E*/*Z* photoisomerization at air–water interfaces, we performed vibrational sum-frequency generation (SFG) spectroscopy. Note, that SFG is a highly interface-specific spectroscopic method that can provide molecular-level details of interfaces and is explained in more detail in ESI.† SFG spectroscopy has been used to understand the role of polymers in inhibiting precipitation and enhancing drug solubility for supersaturated drug delivery systems.^28^ The chemistry of polymer-nanoparticle composites was also probed using SFG for controlled drug delivery applications.^29^ Wang *et al.* combined SFG with a microfluidic flow cell to investigate molecular level interactions between a polymeric drug and model cell membranes.^30^ In Fig. 2, we present the vibrational SFG spectra of AAP, Dox, and AAP–Dox mixtures at the air–water interface. The SFG spectra of air–water interfaces in the absence of Dox and at AAP bulk concentration of 0.3 mM for both *E* and *Z* isomers are shown in Fig. 2a. Vibrational bands from the alkyl chain of the AAP surfactant dominate the spectrum of *E*-AAP, with the CH_3_ deformations at ∼1400 cm^−1^ and CH_2_ bending vibrations centred at ∼1430 cm^−1^.^31^ The bands at 1550 and 1600 cm^−1^ originate from the C=C stretching vibrations of the aromatic rings of the AAP.^32^ We see that *E* → *Z* photoisomerization leads to an overall decrease in the intensity of the vibrational bands at the air–water interface because of the increased hydrophilicity and, thus, decreased surface-activity of the *Z*-isomer. This observation is consistent with our results from the compression isotherms (ESI†) which show large changes in the surface activity of the *E*- and *Z*-AAP. The SFG spectrum of 5 μM Dox (Fig. 2b) without AAP at the air–water interface shows a characteristic band around 1580 cm^−1^ which is attributable to the C=C aromatic stretching vibration of Dox.^33^ Next, we focus on the light-induced changes at the air–water interface for Dox–AAP solutions. In all the solutions, the concentration of Dox was fixed to 5 μM. When the Dox–AAP mixtures were irradiated by green light with the predominant *E* isomer of AAP, vibrational bands at 1400, 1430, 1550, 1600 and 1580 cm^−1^ in the SFG spectra (Fig. 2c and e) suggest the presence of both Dox and AAP molecules at the interface. As we will discuss in more detail below the absence of the 1580 cm^−1^ band in Fig. 2d (0.3 mM AAP) is indicative for fully encapsulated Dox molecules in bulk *E*-AAP micelles and the interface is dominated by free *E*-AAP moieties.

Upon UV irradiation the AAP surfactants undergo *E* → *Z* photoisomerization driving the transition from surfactant micelles of the *E* isomer to free monomers of the *Z* isomer when the AAP concentrations are between >0.1 and <1 mM. As a result, Dox can be released from the micelles and adsorb to the interface where the less-surface active AAP *Z*-isomer canpartially desorb from the interface. Indeed, we observe a substantial decrease in the intensity of the vibrational bands centred at 1400, 1430, 1550 and 1600 cm^−1^ due to interfacial AAP suggesting photoisomerization-induced interfacial restructuring and desorption. However, the intensity of the vibrational band at 1580 cm^−1^ corresponding to Dox at the interface remains either largely unchanged (Fig. 2c) or even increases (Fig. 2d) depending on the AAP–Dox mixing ratio. In fact, when the AAP concentration was 0.3 mM, the SFG spectrum of the *E*-AAP–Dox solution showed bands corresponding to molecular vibrations in AAP but no apparent peak at 1580 cm^−1^. The absence of a vibrational band at 1580 cm^−1^ clearly shows that the coverage of free Dox moieties at the air–water interface is neglectable and, thus, encapsulation in the AAP micelles at 0.3 mM is highly efficient to prevent free (unbound) Dox moieties from entering the interface as long as the AAP surfactants are in the predominant *E*-state. However, a strong vibrational band at 1580 cm^−1^ was observed when the samples were subjected to UV light causing *E* → *Z* photoisomerization of the surfactant as well as a subsequent and substantial release of free Dox moieties which readily adsorb to the interface. We lastly comment on the light-induced changes at the interface at 1.2 mM AAP which is a concentration above the CMC of both *E*- and *Z*-AAP. In Fig. 2e, clearly, SFG signals corresponding to interfacial AAP are visible for *E*-AAP whereas a decrease in intensity upon irradiation from green to UV light and thus for predominant *Z* isomers is seen and is accompanied by the appearance of a weak band centred at 1580 cm^−1^ due to C=C stretching vibration of free interfacial Dox molecules. The low intensity of the vibrational band attributable to Dox molecules at the interface for both isomers of the AAP is suggestive of the encapsulation of the majority of the Dox molecules in the micelles of both the *E*- and *Z*-isomers as long as the concentration is above the CMC for both isomers (>1 mM).

**Fig. 3:**
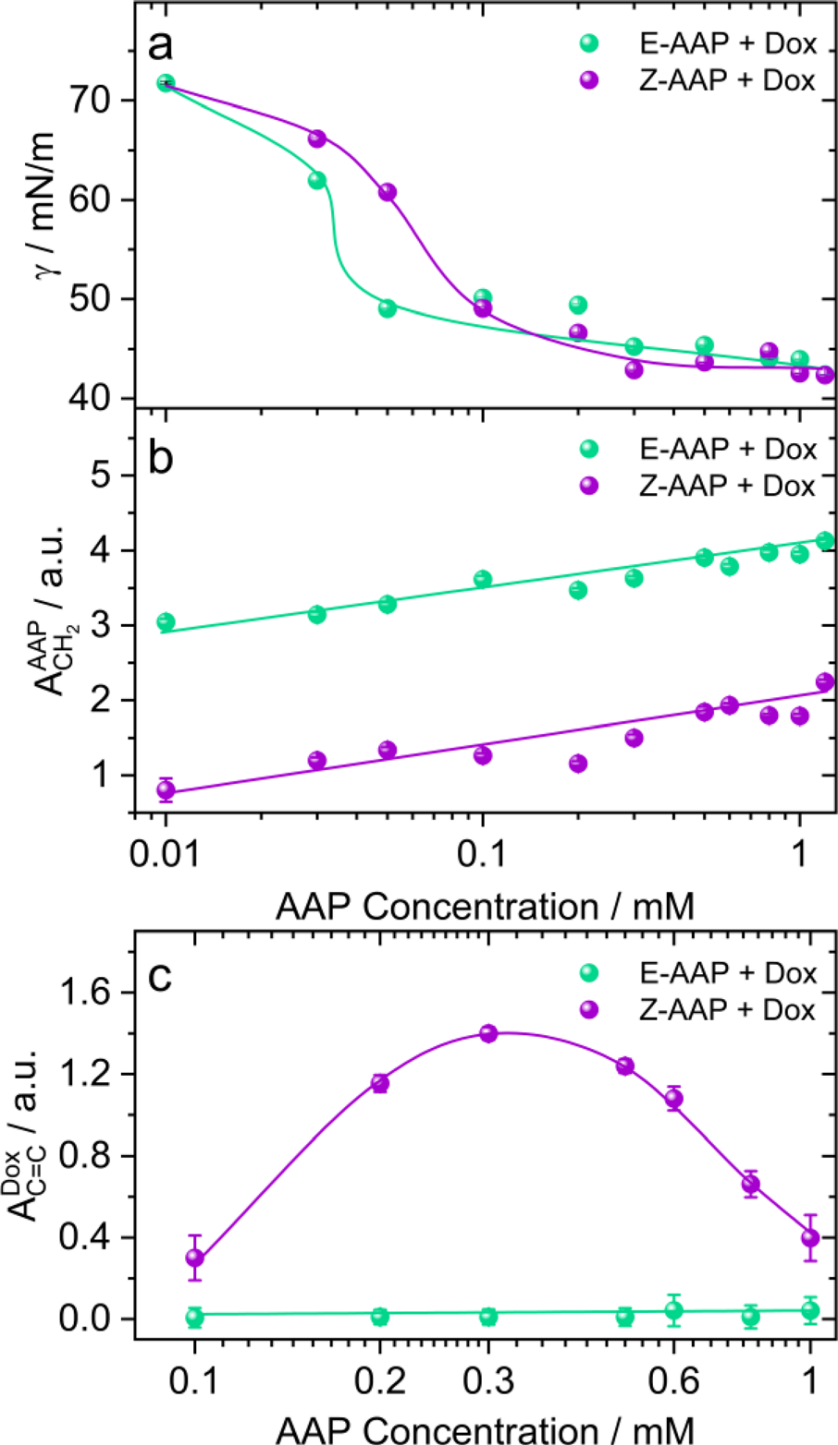
(a) Equilibrium surface tension *γ* and SFG amplitudes of (b) CH_2_ bending vibrations of AAP (∼1430 cm^−1^) and (c) C=C aromatic stretching vibrations of Dox (∼1580 cm^−1^) of AAP–Dox mixtures at the air–water interface as a function of AAP concentrations. Results for the *E*-AAP isomer are shown as green spheres whereas results for *Z*-AAP are shown as violet spheres. Results were measured for irradiation with (520 nm, 3 mW cm^−2^) green and (365 nm, 10 mW cm^−2^) UV light, respectively. The equilibrium surface tension values were noted after 1 h of adsorption at the air–water interface. The amplitudes are obtained from the SFG spectra recorded after 30 min of green/UV light illumination. The concentration of Dox was fixed to 5 μM. The solid lines in green and violet colours guide the eye.

To address the effect of AAP concentration on the photoisomerization-induced changes in the interfacial structure, we recorded the equilibrium surface tension of Dox–AAP solutions as a function of AAP concentration that was varied from 0.01 to 1.2 mM (Fig. 3a). Hardt *et al.* have previously shown that *Z*-AAP is less surface-active resulting in a higher equilibrium surface tension compared to the more hydrophobic *E* isomer as long as the concentrations are below the CMC of *Z* isomer.^23^ At this point, we would also like to emphasize on the Δ*γ*_max_ which is the maximum change in the equilibrium surface tension upon *E*–*Z* photoswitching. Previously, it was observed that Δ*γ*_max_ for the AAP surfactant was 23 mN m^−1^ at 0.1 mM AAP without Dox.^23^ For AAP–Dox mixtures Δ*γ*_max_ is significantly lower (Fig. 3a), which is indicative of the presence of free Dox moieties at the interface and is consistent with results from SFG spectroscopy that demonstrate a release of Dox from *E*-AAP micelles when the surfactants photoisomerize from the *E* to the *Z* isomer (Fig. 2 and S4†). It is important to mention that Dox in the absence of AAP does not significantly reduce the surface tension of water.

In Fig. 3b, we compare the SFG amplitudes of CH_2_ bending vibration bands of AAP at ∼1430 cm^−1^ as a function of AAP concentration in the Dox–AAP solutions. The amplitudes of the C–H bending vibrations of *E*-AAP show a steady increase with the AAP concentration. Under UV irradiation, we see lower SFG amplitudes for the less-surface active but more hydrophilic *Z*-AAP. This can be easily ascribed to the increase in interfacial coverage by AAP molecules with concentration.

Next, we compared the extent of Dox release as a function of AAP concentration for AAP–Dox mixtures. We would like to recall that the concentration range of 0.1 to 1 mM AAP is most relevant to investigate the drug release because at these concentrations, the *E*-isomers of the AAP surfactants are predominantly aggregated as micelles, whereas the *Z*-isomers only form micelles at concentrations ≥1 mM. Fig. 3c shows the SFG amplitudes of the aromatic stretching of Dox at the air–water interface for different Dox–AAP mixing ratios. We note a significant enhancement in the SFG amplitudes of Dox bands at these concentrations presumably because the *E*-AAP is predominantly in micelles encapsulating most of the Dox molecules in the Dox–AAP solution. The exposure to UV light disrupts the micellar self-assembly and the encapsulated Dox molecules are released to the interface as long as the concentration of *Z*-AAP in the solution is far below its CMC. We observe that at concentrations of 0.3 mM AAP, the Dox signal is largely enhanced upon UV irradiation indicating a substantial release of Dox from the micelles in the bulk solution to the interface. This is consistent with our proposition that photoisomerization of AAP results in the release of Dox to the interface. Here, we emphasize the importance of capturing Dox release at the air–water interface with vibrational SFG spectroscopy, because only a fraction of the total molecules in the bulk aqueous solution may be able to adsorb at the interface. The appearance of a strong SFG signal for Dox at the air–water interface implies that the drug release to the interface is significant.

In order to gain additional information on the Dox release in the bulk solution, we have performed steady-state fluorescence spectroscopy. This is presented in Fig. S5 in the ESI.† Encapsulation of Dox in the micelles of *E*-AAP leads to a significant quenching of the Dox fluorescence and subsequent exposure to UV irradiation results in an enhancement of the fluorescence. This confirms the UV light-induced release of Dox encapsulated in AAP micelles in the bulk aqueous solution. The quenching of fluorescence upon encapsulation within micellar nanocarriers has been attributed to the effective encapsulation of Dox in the hydrophobic interior of the micelles.^34^ In addition, using large hydrated iodide ions as a quencher, we show that Dox in *Z*-AAP is more exposed to aqueous solution than in *E*-AAP. Hence, this provides conclusive evidence that the *E*/*Z* isomerization of AAP surfactants results in the light-induced release of encapsulated Dox molecules in bulk aqueous solution as well. It is crucial to note that drug release at the air–water interface is closely linked to the release in the bulk aqueous solution. Upon UV irradiation, the AAP micelles in the bulk disassemble, releasing the encapsulated Dox into the bulk solution, as demonstrated in Fig. S5 of the ESI.† However, the appearance of Dox at the air–water interface is only feasible if two conditions are met: (1) the drug possesses inherent surface activity, and (2) the interface is not completely saturated by surfactant molecules. Our results show that both conditions can be satisfied for Dox–AAP micellar nanocarriers, allowing us to observe and capture the release of Dox at the air–water interface. This provides important insights into the drug's interaction with the interface, complementing its release behavior in the bulk phase.

Next, we focus on the characteristic time scales of changes in the interfacial structure accompanying the release of Dox to the air–water interface.

**Fig. 4:**
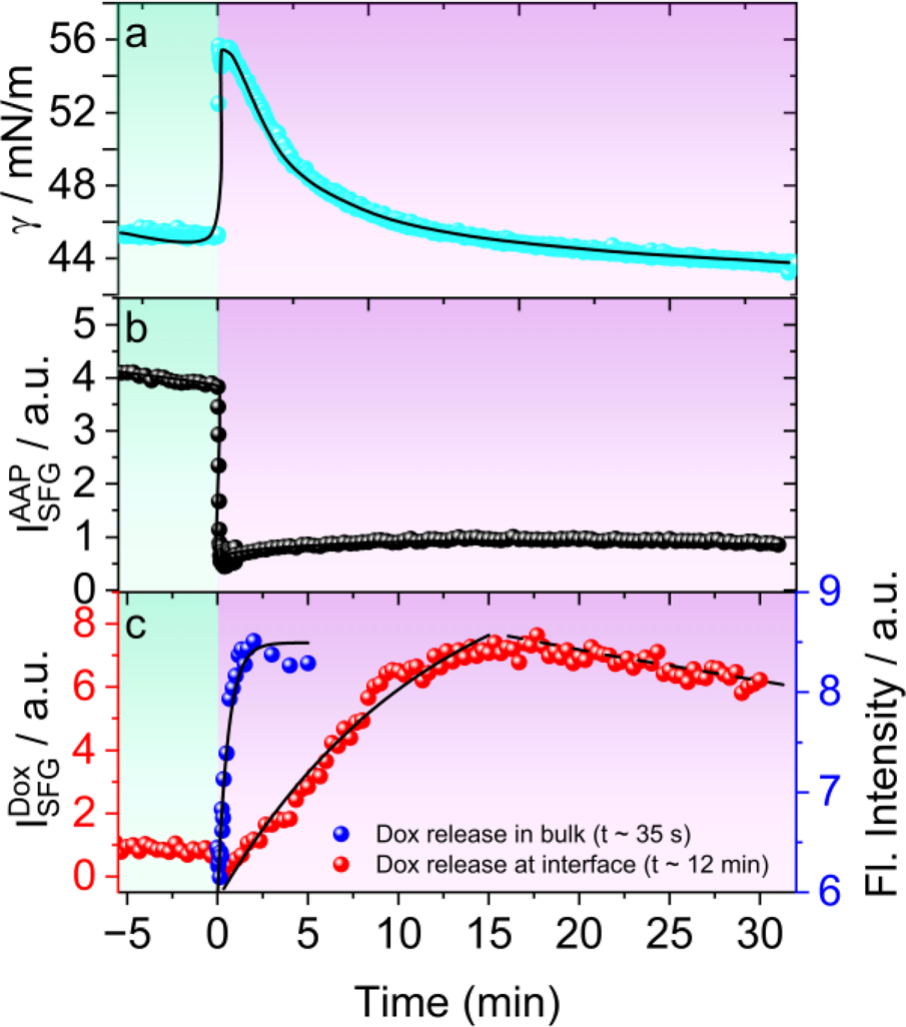
(a) Dynamic surface tension (*γ*, cyan spheres), (b) maximum SFG intensities of CH_2_ bending vibrations of the AAP (*I*^AAP^_SFG_, black spheres) as well as (c) C=C stretching of Dox (*I*^Dox^_SFG_, red spheres) and integrated fluorescence intensities (blue spheres) of the AAP–Dox mixture as a function of time. The concentrations of AAP and Dox were 0.3 mM and 5 μM, respectively. The sample was irradiated with green (520 nm, 3 mW cm^−2^) light for the first 5 min and then switched to UV (365 nm, 10 mW cm^−2^) light for the next 30 min of the measurement (also indicated by the background colour of the plot). The time *t* = 0 indicates the time point when the UV light was turned on. The intensities in (c) were fitted with a first-order kinetics (*I* = *I*_1_(1 − e^−*t*/*t*_0_^) + *I*_0_) indicated by solid black lines in (c). The dashed lines in (c) and the solid black lines in (a) and (b) are drawn as guides to the eye.

For this, we recorded the dynamic changes in the surface tension *γ*(*t*) of 0.3 mM AAP–Dox solution as a function of time upon *E* → *Z* switching which was started by changing the light irradiation at *t* = 0 min. Prior to this experiment, the AAP–Dox solution was equilibrated by prolonged exposure to green light. Switching to UV light, causes a sharp rise in *γ*(*t*) followed by a subsequent slower decrease in surface tension which plateaus at a lower *γ* (Fig. 4a). To understand the molecular origin of this behaviour we have performed time-dependent SFG spectroscopy and recorded SFG spectra as a function of time after initiating *E* → *Z* photoisomerization of the AAP surfactants which are presented in Fig. S6 in the ESI.† In Fig. 4b, we plot the time-dependent changes in the SFG intensity of CH_2_ bending vibrations of AAP at 1430 cm^−1^. A rapid decrease in the SFG intensity of AAP bands suggests a fast desorption of AAP molecules from the air–water interface upon photoisomerization to the more hydrophilic *Z*-isomer. This explains the initial increase in the *γ* within 1 min after UV irradiation was started (Fig. 4a). For tracking the release of free Dox at the air–water interface we are using the SFG intensity of the Dox specific C=C stretching band at 1580 cm^−1^ which is plotted as a function of time in Fig. 4c. Close inspection of Fig. 4c shows a maximum release of Dox at about 15 min and a majority of the Dox molecules stays adsorbed at the interface as long as the UV light is on *i.e.* 30 min. Therefore, the photoisomerization-induced changes in *γ* can be clearly accounted for by the dynamic changes in the molecular structure of the air–water interface. At this point, we compared the kinetics of Dox release in bulk solution by measuring the fluorescence of Dox upon UV irradiation (blue squares in Fig. 4c). Our analysis shows that Dox release occurs much faster in bulk solution (*t*_0_ = 35 s), while the drug release at the interface is considerably slower (*t*_0_ = 12 min). This slower release at the interface is due to the sequential process of Dox being released in the bulk solution, followed by its diffusion to the interface, and subsequent adsorption at the interface. As discussed in the previous section, this adsorption of Dox at the interface is possible because the less surface-active *Z*-AAP molecules desorb from the interface to a large extent, allowing the more surface-active Dox to dominate. Consequently, the interface becomes populated by free Dox molecules, whereas before release, they remained encapsulated in the bulk solution. We also performed experiments at lower intensities of UV irradiation and looked at the Dox release at the air–water interface. Fig. S7† shows the Dox release profiles when the UV light intensity was varied from 0 to 10 mW cm^−2^. The lowest UV intensity at which we clearly observe Dox release to the interface is 2 mW cm^−2^.

At this point, we note that while our experiments were conducted at the air–water interface as a basic model for organic–aqueous interfaces in biological systems, drawing a direct analogy between the two is not straightforward. For instance, pioneering studies by Walker *et al.* have elucidated dramatic effects of the organic layer on the self-assembly of phospholipids at aqueous–organic interfaces.^35^ The presence of an organic solvent led to more expanded phospholipid monolayers in comparison to the air–water interface due to interactions between the lipid acyl chains and the solvent, whereas dispersive and H-bonding interactions can substantially change the interfacial organization.^36^ More recent work by Doughty and co-workers^37^ demonstrated different packing of organophosphorus ligands at oil–water and air–water interfaces, which arise from the solvation of the ligands (tails and heads) on both sides of the liquid–liquid interface which is necessarily not the case for air–water interfaces, where *i.e.* surfactant tails interact only with their nearest neighbors. Investigating drug release at interfaces of two immiscible liquids can be more complex and challenging than at air–aqueous interfaces because the composition of the organic layer tends to influence the self-assembly, organization and transport^38^ through an interfacial layer greatly. To predict the drug release at organic–aqueous interfaces, we must understand that the adsorption of a surface-active molecule from the bulk aqueous solution to the interface can involve the displacement and interaction with other *e.g.* organic moieties at the interface. Therefore, drug release at organic–aqueous interfaces would also depend strongly on the polarity of the organic layer.^37,39^

## Conclusions

In conclusion, we have applied a photoswitchable AAP surfactant as a micellar nanocarrier for the capture and release of doxorubicin within several minutes at air–water interfaces using a relatively low intensity UV irradiation (≤10 mW cm^−2^; Fig. S7†). Our investigation extends beyond conventional bulk aqueous solutions to explore the drug release at the air–water interface, a critical aspect often overlooked in previous studies. A combination of complementary surface-sensitive techniques such as Langmuir–Blodgett technique, surface tensiometry, and vibrational SFG spectroscopy provided valuable insights, demonstrating the release of encapsulated Dox to the air–water interface. These findings contribute a fundamental perspective to the understanding of drug release at hydrophobic interfaces.

Time-dependent SFG measurements revealed that the encapsulated Dox in *E*-AAP micelles is released to the interface along with the *Z*-AAP monomers. Particularly, we have demonstrated that the drug release can be tuned by adjusting the system to switch from a state where the drug molecules are encapsulated in the bulk to a state where the interface becomes dominated by drug molecules. From our results, we can clearly distinguish the kinetics of drug release between the bulk solution and the interface, revealing that the interplay between bulk release and interfacial adsorption is a critical factor in controlling drug release at the point of care, which could be a hydrophobic interface.

Moreover, studies aimed at elucidating drug–carrier interactions at interfaces might offer new insights for further optimization in the field of nanocarrier design for drug delivery.

## Author contributions

I. P. and B. B. conceived the project. M. H. performed the synthesis and characterization of the surfactant. D. G. helped with the initialization of the experiments. B. B. did the funding acquisition and overall project administration. I. P. and B. B. wrote the manuscript and all authors approved the final version of the manuscript.

## Conflicts of interest

There are no conflicts to declare.

## Supplementary Material

SC-015-D4SC04837G-s001

## Data Availability

The data that support these findings of this article are available in the ESI,† while raw data can be made available on request from the corresponding author.
