# Supplementary material for: Photo-induced drug release at interfaces with arylazopyrazoles
Source: Chem Sci. 2024 Oct 17;15(45):18865–71. doi: 10.1039/d4sc04837g (PMC11503751; doi:10.1039/d4sc04837g)
Supplement: SC-015-D4SC04837G-s001 [file SC-015-D4SC04837G-s001.pdf]

Supplementary Information (SI) for Chemical Science.
This journal is © The Royal Society of Chemistry 2024

ELECTRONIC SUPPLEMENTARY INFORMATION

Photo-induced drug release at interfaces with arylazopyrazoles

Ipsita Pani, Michael Hardt, Dana Glikman and Björn Braunschweig*

Institute of Physical Chemistry, University of Münster, Corrensstraße 28-30, 48149 Münster, Germany,
Center for Soft Nanoscience (SoN), University of Münster, Busso-Peus-Straße 10, 48149 Münster, Germany.
E-mail: braunschweig@uni-muenster.de

1. Materials and methods 2

2. UV-Vis Spectroscopy of AAP 4

3. Langmuir compression isotherms 5

4. SFG spectroscopy of AAP-Dox mixtures 6

5. Steady-state fluorescence measurements 6

6. Time-dependent SFG Spectroscopy of AAP-Dox mixtures 8

6. References 8

1. Materials and methods

Materials Doxorubicin hydrochloride was purchased from BLD Pharmatech GmbH and was used as received. Detailed synthesis and characterization of the AAP surfactant (octyl-arylazopyrazole butyl sulfonate) is reported elsewhere.[1,2] Potassium iodide (99% purity) was purchased from Acros Organics. All glassware used in this study were systematically cleaned, first with Alconox solution (Sigma-Aldrich), then immersed in an acid bath (a solution of concentrated sulfuric acid and Nochromix) for atleast 16 h and finally rinsed with ample amount of ultrapure water from a Merck MilliQ Reference A+ purification system. Calculated amount of AAP and Dox were weighed and dissolved in MilliQ water to prepare stock solutions of 5 mM. The stock solutions were then diluted to the desired concentrations.

UV-Visible Spectroscopy Ultraviolet-Visible (UV-vis) absorption spectra were recorded with a Perkin Elmer LAMBDA 650 UV/vis spectrometer in the wavelength range of 250-700 nm. The absorption spectrum for E-AAP was recorded after continuous irradiation of aqueous solution of AAP with 520 nm green light (4 $mWcm^{-2}$) for 10 min. After that the sample was irradiated with 365 nm UV light (10 $mWcm^{-2}$) for different durations and the spectra were recorded.

Compression isotherms The surfactant monolayers were prepared using a KSV Nima Langmuir-Blodgett trough (Biolin Scientific, Sweden) equipped with two hydrophilic barriers. AAP was weighed and dissolved in $CHCl_3$ (98%) to form a light-yellow suspension of concentration 1 mg/mL. 250 and 150 µL of E- and Z-AAP solutions were spread on the Langmuir trough containing water/Dox. The chloroform was allowed to evaporate for 5-10 min and the barriers were compressed symmetrically at a rate of 20 mm/min. A filter paper Wilhelmy plate placed at the center of the Langmuir trough measured the surface pressure. During the experiment, the Langmuir trough was illuminated centrally with green (520 nm, 4 $mWcm^{-2}$) or UV (365 nm, 4 $mWcm^{-2}$) light with the LED placed at a distance of approximately 5 cm from the trough using a custom-designed holder for the LEDs. The intensities of the LEDs were measured with the help of a power meter from Thor Labs. The compression isotherms at each concentration of Dox were recorded at least 3 times. The compressibility modulus was calculated for each isotherm using the following equation,

$$C_S^{-1} = -A\frac{d\pi}{dA} \quad (1)$$

where C_S^{-1} is the compressibility modulus, π is surface pressure (mN/m), A is mean molecular area ($Å^2$). The average and standard deviation of the limiting mean molecular area, final surface pressure and the compressibility modulus were calculated from 3 independent measurements.

Surface tension measurements Stock solutions of AAP and Dox were prepared by dissolving AAP and Dox in milliQ water (total oxidizable Carbon ≤ 3ppm). Calculated volumes of stock solutions of AAP (5 mM) and Dox (5 mM) were mixed and stirred for 2 h to prepare Dox-AAP solutions. Dox concentration was fixed to 5 µM and the AAP concentration was varied from 0.01 – 1.2 mM. The solution was stirred at 350 rpm over a water bath with temperature maintained at 60 ℃ for 90 min. Surface tension of the Dox-AAP solutions were measured at air-water interface using a pendant drop tensiometer PAT1M by analysis of the drop shape using Young Laplace equation. The dynamic surface tension was measured for 1 h under green (520 nm) and UV (365 nm) light. The equilibrium surface tension was calculated as an average of the surface tension values from the last 20 values of reaching equilibrium.

Vibrational Sum Frequency Generation (SFG) Spectroscopy Vibrational SFG spectroscopy is a powerful technique to study the molecular structure of interfaces. In SFG spectroscopy, a sum frequency beam (SF)

is generated by the overlap of a frequency-tunable infrared (IR) beam and a fixed-frequency visible (VIS) beam ($\omega_{SF} = \omega_{IR} + \omega_{VIS}$). The frequency of the IR beam can be tuned to excite the desired molecular vibrations from the ground to an excited state. The SFG intensity can be described by the following equation,[1,3]

$$I_{SF} \propto \left|\chi_{NR}^{(2)} + \sum_q \frac{A_q}{\omega_{IR}-\omega_q+i\gamma_q}\right|^2 I_{IR}\, I_{VIS} \qquad (2)$$

where, $\chi_{NR}^{(2)}$ is the non-resonant contribution to second order electric susceptibility while the second term is the resonant contribution to the effective second-order electric susceptibibilty $\chi_{eff}^{(2)}$. The resonant contributions to the SFG signal are a function of the resonance frequency of the molecular vibration ω_q, frequency of the IR beam ω_{IR}, bandwidth of the vibrational mode Γ_q, and the oscillator strength A_q, respectively. The term $A_q \propto \Gamma_q \left\langle\beta_q^{(2)}\right\rangle$ has important implications on the SFG signal as it depends on the surface excess of the interfacial molecules Γ_q, the orientational average $\langle\ldots\rangle$ of the molecular hyperpolarizability $\beta_q^{(2)}$. The dependence of the SFG signal on the orientational average has interesting consequences. For bulk phases with inversion symmetry like in aqueous electrolytes, isotropic liquids and gas, the orientational average of $\beta_q^{(2)}$ is necessearily zero and bulk contributions to the SFG signal are, thus, symmetry forbitten. Interfaces like the air-water interface, however, break the bulk inversion symmetry and, consequently, give rise to new contributions to the second-order electric susceptibility rendering SFG spectroscopy inherently interface-specific.

In this work, we have used a 1 kHz broadband IR-vis SFG spectrometer that was described in detailed previously.[3] In brief we have used frequency-tunable 70 fs IR pulses with a bandwidth of >300 cm^{-1} and a pulse energy of 12 µJ that are combined with 1 to 2 ps 'visible' pulses with a wavelength of 804.1 nm, a bandwidth of <5 cm^{-1} and a pulse energy of 25 µJ. The IR and the 'visible' pulses were overlapped at the sample at angles of 60° and 55° vs the surface normal. The SFG beam reflected from the sample was guided to a spectrograph (Kymera, Andor UK) and detected with an EMCCD (Newton, Andor UK). The recorded spectra were normalized by the SFG spectrum of a plasma-cleaned Au substrate which we have used as a reference. All spectra at air-water interface were recorded using p-polarized IR, s-polarized visible and s-polarized SF beams. For recording the SFG spectra, 3.5 mL of the Dox-AAP solutions were pipetted on to glass petridishes and placed on an automized sample holder. Prior to their use in SFG spectroscopy, the glass petridishes were kept immersed in an acid bath (solution of sulphuric acid and Nochromix) for atleast 12 hours, rinsed with ample amount of MilliQ water and dried with a stream of N_2 gas before the experiment. The samples were irradiated *in situ* with green (520 nm, 4 $mWcm^{-2}$) and UV (365 nm, 10 $mWcm^{-2}$) using LEDs inside the sample chamber during the measurement.

Fluorescence spectrometry Calculated volumes of stock solutions of AAP (5 mM) and Dox (5 mM) were mixed a round bottom flask to have final concentrations of 0.3 mM AAP and 5 µM Dox. This solution was stirred for 2 h to ensure the encapsulation of Dox in AAP micelles. The solution was kept in dark for at least 2 h before fluorescence measurements. The Dox-AAP solutions were then transferred to 3 mL glass cuvettes and irradiated with green (520 nm, 3-4 $mWcm^{-2}$) or UV (365 nm, 10 $mWcm^{-2}$) light for 5 min before measurements. All the fluorescence emission spectra were recorded using a FP 8500 spectrofluorometer (JASCO). For the KI quenching experiments, AAP-Dox solutions (0.3 mM AAP, 5 µM Dox) with KI concentrations varying from 0-1.66 mM were prepared and the spectra were recorded.

2. UV-Vis Spectroscopy of AAP

The UV-vis spectra of the E- and Z- AAP are shown in Fig. S1. The absorption spectrum of E-AAP (recorded after 10 min irradiation with 520 nm green light) shows a strong $\pi \rightarrow \pi^*$ band at 340 nm and a weak $n \rightarrow \pi^*$ band at ~ 426 nm. The absorption spectra of the E-AAP (after UV irradiation for ≥ 1 min) show a blue-shifted $\pi \rightarrow \pi^*$ band at 304 nm and a red-shifted $n \rightarrow \pi^*$ band at 436 nm.

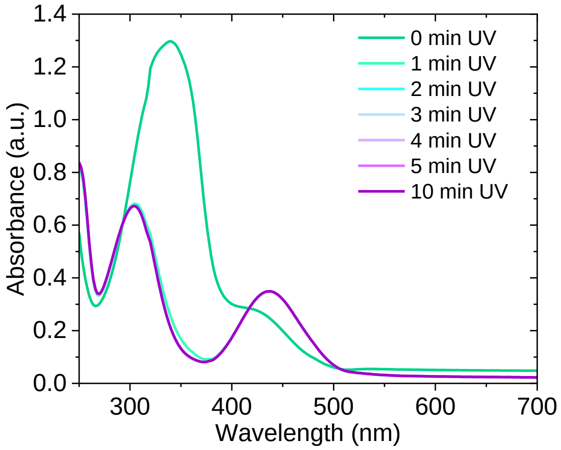

Fig. S1. UV/vis spectra of aqueous solution of 0.1 mM AAP after irradiation with 10 min of 520 nm green light (indicated as 0 min UV) and different irradiation times with 365 nm UV light as indicated in the figure. Note, already after 1 min UV irradiation equilibrium is reached and the spectra overlap with the final spectrum at 10 min without noticeable changes.

3. Compression Isotherms

In the absence of Dox, both E- and Z-AAP form compact monolayers at the air-water interface. However, the value of final surface pressure of the Z-AAP is ~ 3 times lower than the E-AAP. This could be attributed to the low surface activity of the Z-isomer. An additional contributing factor could be the desorption of some of the surfactant molecules from the interface to the aqueous subphase because of the increased hydrophilicity of the Z-isomer. A close inspection of the surfactant isotherms with Dox in the aqueous subphase reveals 3 striking features. First, presence of Dox in the aqueous subphase leads to an overall expansion of the monolayers suggesting the incorporation of Dox into the surfactant monolayer. Second, the collapse pressure of the isotherms increases with the concentration of Dox suggesting the presence of Dox in the monolayer even at higher surface pressure values. Third, at 8 µM Dox, we note that the surface pressure values decrease (at about 30 mN/m for E-AAP, Fig. S2a) or remain constant (at about 25 mN/m for Z-AAP, Fig. S2b) as the molecules get closer. This can be attributed to the partial expulsion of Dox and/or AAP molecules from the air-water interface to the aqueous phase. Similar features have been noted in the isotherms of membrane lipids when spread over an aqueous subphase of Dox.[4–6] In order to look at the changes in the surface packing of the AAP in presence of Dox quantitatively, we calculated the inverse of compressibility modulus C_s^{-1}. The C_s^{-1} is a measure of the compactness of a monolayer. The plots of C_s^{-1} calculated from Fig. S2a and b are shown in Fig. S2c and d. We note that the maximum values of C_s^{-1} increase with the concentration of Dox. However, the change in values is more significant for the Z-AAP. We have compared the limiting mean molecular area, maximum surface pressure and maximum C_s^{-1} for both the isomers in Fig. S3.

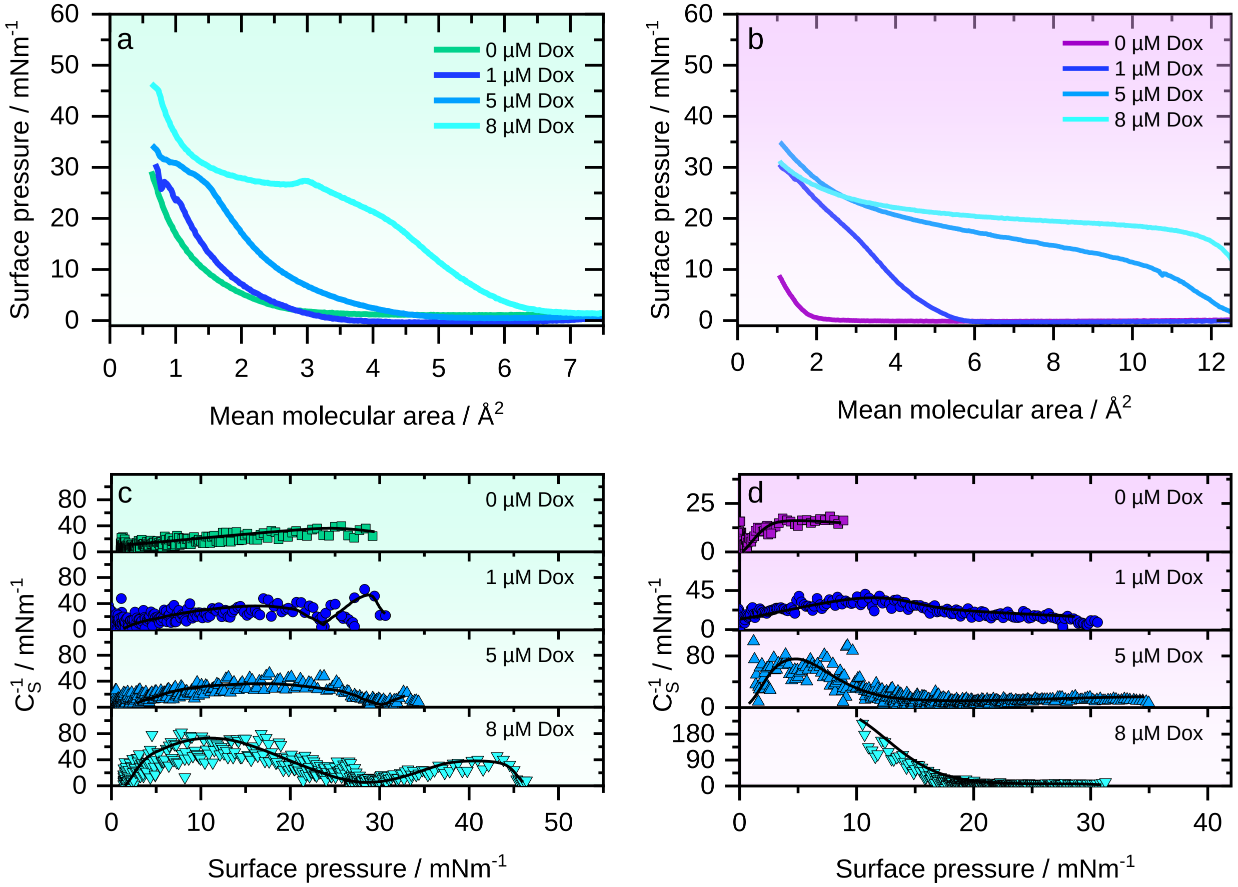

Fig. S2 Compression isotherms of (a) E-AAP and (b) Z-AAP on aqueous subphases containing 0, 1, 5 and 8 µM Dox. Compressibility modulus vs. surface pressure plots of (c) E-AAP and (d) Z-AAP calculated from the isotherms shown in (a) and (b), respectively. The scatter points denote the values of the compressibility modulus and the solid lines are drawn as guides to the eye. The isotherms for E- and Z-AAP were recorded under illumination with green (520 nm, 4 mWcm^{-2}) and UV light (365 nm, 4 mWcm^{-2}), respectively.

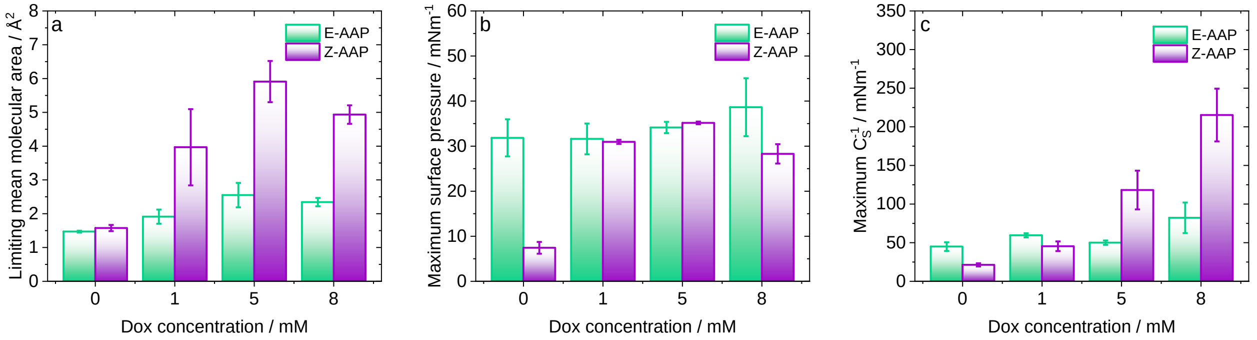

Fig. S3. Histograms showing mean values of the (a) limiting mean molecular area, (b) maximum surface pressure and (c) maximum compressibility modulus of the Langmuir monolayers of E- and Z-AAP on aqueous subphases containing 0, 1, 5 and 8 µM Dox. The errors bars show the standard deviation calculated from 3 sets of measurements.

4. SFG spectroscopy of AAP-Dox mixtures

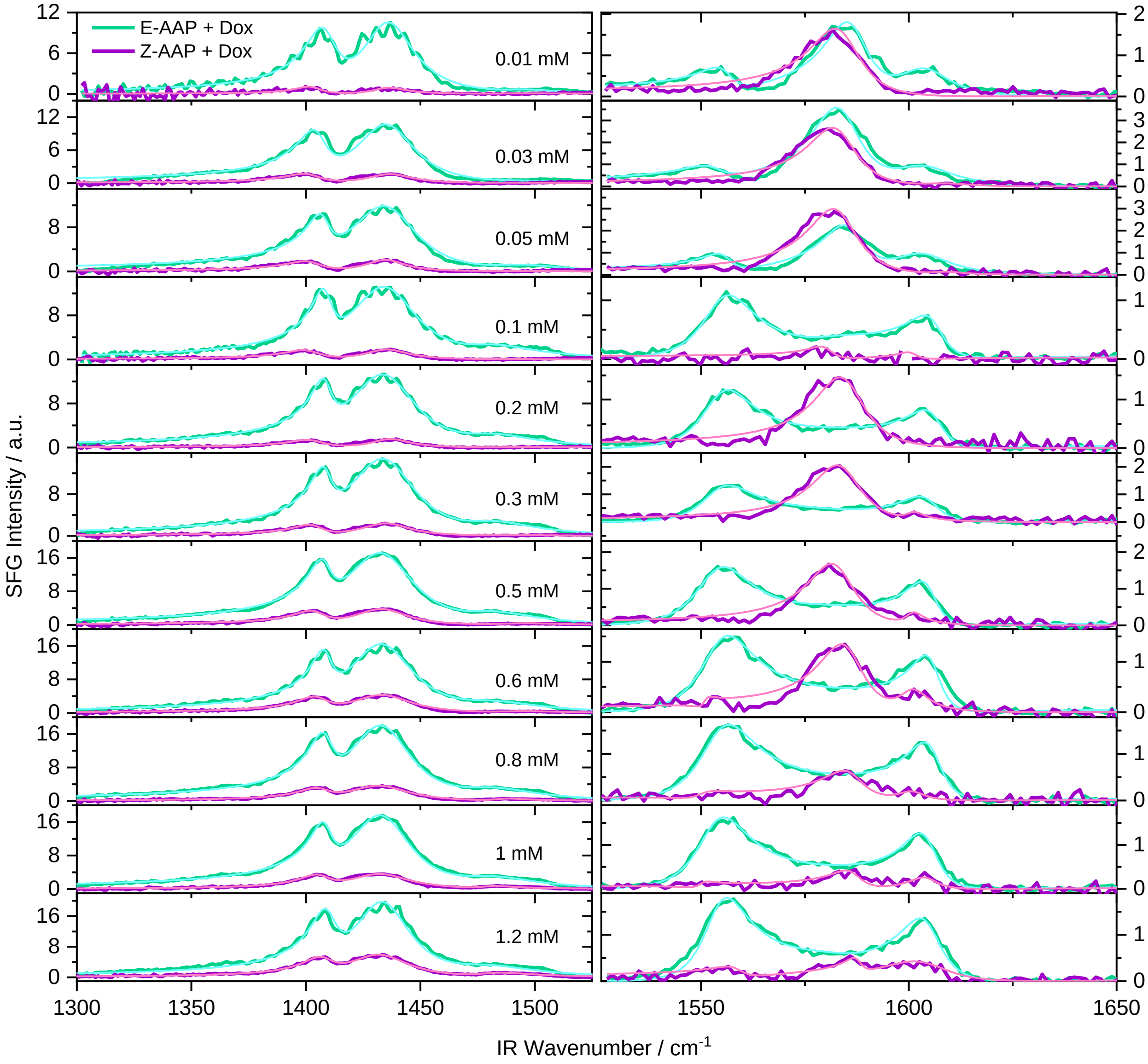

Fig. S4. SFG spectra of the Dox-AAP mixtures at varying concentrations of AAP. The concentration of Dox was fixed at 5 μM. Fits to the spectra are shown in cyan (E-AAP + Dox) and pink (Z-AAP + Dox).

Table S1. Fit parameters from the analysis of SFG spectra of AAP-Dox mixtures exemplary for 0.3 mM AAP + 5µM Dox mixture.

Molecular vibrations	Fit parameters	**E-AAP + Dox**	**Z-AAP + Dox**
CH_3 deformations (AAP)	X_{NR}	0.3	0.3
	A_q / a.u.	1.4	0.8
	Γ_q / cm^{-1}	5.6	6.1
	φ / π	1	1
	ω_q / cm^{-1}	1408	1406
CH_2 bending (AAP)	X_{NR}	0.3	0.3
	A_q / a.u.	3.6	1.5
	Γ_q / cm^{-1}	18.9	13.1
	φ / π	1	1
	ω_q / cm^{-1}	1435	1435
	X_{NR}	0.3	0.3
	A_q / a.u.	0.8	0.05
	Γ_q / cm^{-1}	18.8	0.01
	φ / π	1	1
	ω_q / cm^{-1}	1485	1485
Ar C=C (AAP)	X_{NR}	0.2	0.2
	A_q / a.u.	1.1	0.01
	Γ_q / cm^{-1}	7.6	0.3
	φ / π	0	0
	ω_q / cm^{-1}	1554	1554
Ar C=C (Dox)	X_{NR}	0.2	0.2
	A_q / a.u.	0.01	1.4
	Γ_q / cm^{-1}	6.1	7.4
	φ / π	1	1
	ω_q / cm^{-1}	1584	1584
Ar C=C (AAP)	X_{NR}	0.2	0.2
	A_q / a.u.	0.8	0.3
	Γ_q / cm^{-1}	5.7	2.7
	φ / π	1	1
	ω_q / cm^{-1}	1605	1605

5. Steady-state fluorescence measurements of Dox in water

Dox in water shows a strong fluorescence emission around 600 nm (Fig. S5). Encapsulation of Dox in the micelles of E-AAP leads to a significant quenching of the fluorescence as shown in Fig. 6a. The quenching of fluorescence upon encapsulation within micellar nanocarriers has been attributed to effective encapsulation of Dox in the hydrophobic interior of the micelles.[7] Thus, our observation of a dramatic quenching of Dox in AAP micelles is suggestive of encapsulation of Dox in the hydrophobic micellar core. We noted an enhancement in the fluorescence signal after exposure to UV light for 5 min. The increase in fluorescence intensity indicates that the Dox is now more exposed to water after UV irradiation. This confirms the UV light-induced release of Dox encapsulated in AAP micelles in the bulk aqueous solution.

To gain deeper insights into the light-induced changes in the micellar localization of Dox, we carried out iodide quenching experiments. The hydrated iodide ion is an efficient quencher with a large hydration sphere. Due to its large size, it can only access the fluorophores that are exposed to the aqueous phase and cannot quench effectively if the fluorophore is buried in the hydrophobic micro-environment. We sought to compare the relative localization of Dox in the E- and Z-AAP. The ratio of fluorescence intensity of Dox at different KI concentrations to the initial intensity was plotted as a function of KI concentration for Dox in E-AAP and Z-AAP, respectively. We analysed the drug-quencher interactions in the nanoscopic region of AAP micelles. The extent of quenching is a measure of the aqueous exposure of Dox to the quencher i.e. iodide ions. The lower extent of fluorescence quenching for Dox in E-AAP indicates that the drug is less accessible to the hydrated quencher and is present in the hydrophobic domains of the AAP micelles. Increase in the quenching of Dox fluorescence for Z-AAP indicates that some of the entrapped Dox is now released and is more accessible to the quencher iodide ions. Hence, this provides conclusive evidence that E-Z isomerization of AAP results in the light-induced release of encapsulated Dox in bulk aqueous solution.

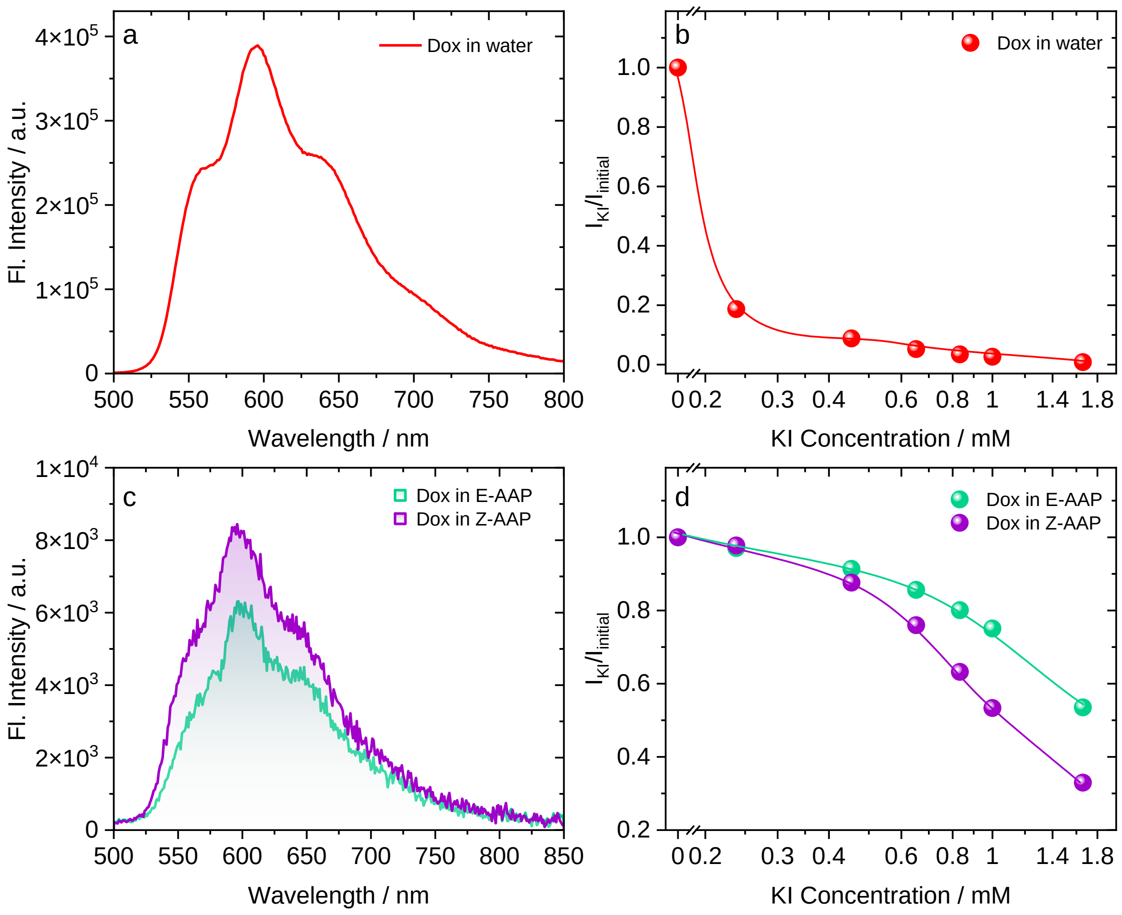

Fig. S5. (a,c) Fluorescence emission spectra of 5 μM Dox in water, E-AAP and Z-AAP. (b,d) Plots showing fluorescence quenching of Dox by iodide ions in water, E-AAP and Z-AAP.

6. Time-dependent SFG spectroscopy

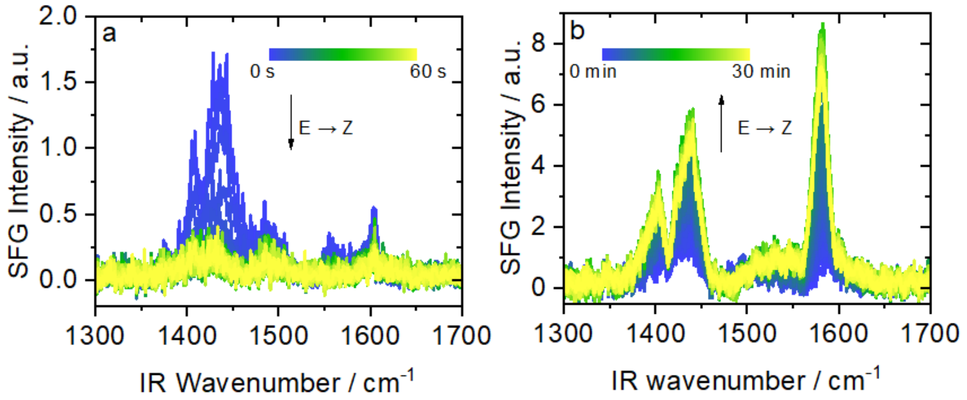

Fig. S6. Time-dependent SFG spectra for E-Z photoisomerization of AAP-Dox mixtures after changing the light illumination from green to UV at t = 0 s at a rate of a) one spectrum per s and b) one spectrum per 20 s.

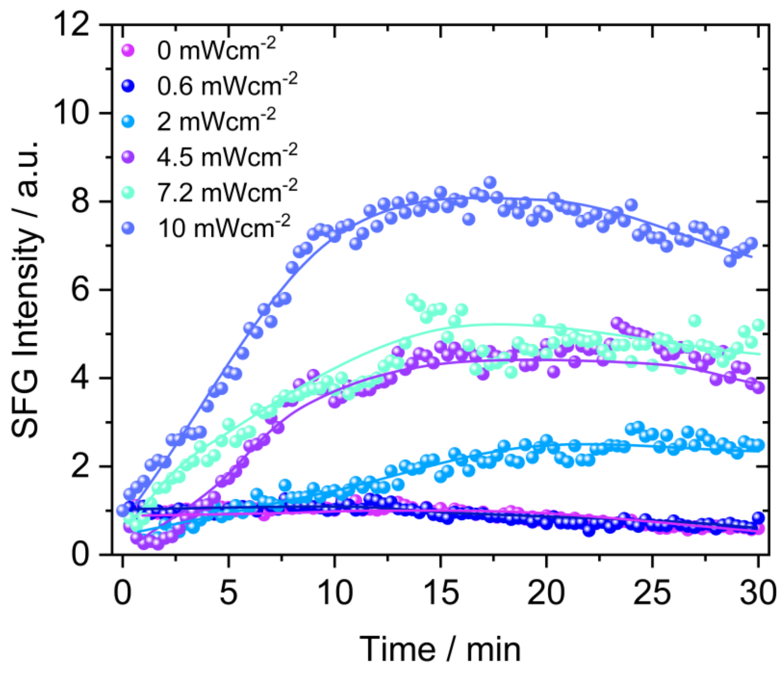

Fig. S7. Time-dependent release of Dox from the AAP-Dox micelles (0.3 mM AAP; 5 µM Dox) to the air-water interface as evidenced from the intensity of the specific Dox related band in SFG spectra at different intensities of UV irradiation. The irradiation of the samples was changed from green to UV at time t=0 min.

Fig. S7 shows the Dox release profiles when the UV light intensity was varied from 0 to 10 $mWcm^{-2}$. The lowest UV intensity at which we clearly observe release of Dox to the interface is 2 $mWcm^{-2}$. However, the kinetics of the release to the interface are slower with lower intensities whereas the maximum coverage of Dox at the interface increases with UV light intensity. Clearly, we can observe a drug release

with UV light intensities close to the maximum permissible exposure of the human skin (~8.3 mW/cm² for 2 min). The results also show that the efficiency likely correlating with the penetration depth of the UV light is dependent on the light as for lower intensities a smaller number of bulk molecules can be affected due to a shorter penetration depth of the UV irradiation into the bulk solution. Therefore, we used the discussion in the main text results for 10 $mWcm^{-2}$ as a tradeoff to understand the Dox release and accompanying kinetic as well as structural changes at the air-water interface upon E-Z photoisomerization.

References

1 C. Honnigfort, R. A. Campbell, J. Droste, P. Gutfreund, M. R. Hansen, B. J. Ravoo and B. Braunschweig, Unexpected monolayer-to-bilayer transition of arylazopyrazole surfactants facilitates superior photo-control of fluid interfaces and colloids, *Chem. Sci.*, 2020, **11**, 2085–2092.

2 M. Hardt, F. Busse, S. Raschke, C. Honnigfort, J. Carrascosa-Tejedor, P. Wenk, P. Gutfreund, R. A. Campbell, A. Heuer and B. Braunschweig, Photo-Responsive Control of Adsorption and Structure Formation at the Air-Water Interface with Arylazopyrazoles, *Langmuir*, 2023, **39**, 5861–5871.

3 N. García Rey, E. Weißenborn, F. Schulze-Zachau, G. Gochev and B. Braunschweig, Quantifying Double-Layer Potentials at Liquid-Gas Interfaces from Vibrational Sum-Frequency Generation, *J. Phys. Chem. C*, 2019, **123**, 1279–1286.

4 D. Nieciecka, A. Królikowska, A. Joniec and P. Krysinski, Partitioning of doxorubicin into Langmuir and Langmuir–Blodgett biomimetic mixed monolayers: Electrochemical and spectroscopic studies, *J. Electroanal. Chem.*, 2013, **710**, 59–69.

5 D. Matyszewska, E. Nazaruk and R. A. Campbell, Interactions of anticancer drugs doxorubicin and idarubicin with lipid monolayers: New insight into the composition, structure and morphology, *J. Colloid Interface Sci.*, 2021, **581**, 403–416.

6 J. A. Ceballos, S. Jaramillo-Isaza, J. C. Calderón, P. B. Miranda and M. A. Giraldo, Doxorubicin Interaction with Lipid Monolayers Leads to Decreased Membrane Stiffness when Experiencing Compression-Expansion Dynamics, *Langmuir*, 2023, **39**, 8603–8611.

7 D. Missirlis, R. Kawamura, N. Tirelli and J. A. Hubbell, Doxorubicin encapsulation and diffusional release from stable, polymeric, hydrogel nanoparticles, *Eur. J. Pharm. Sci.*, 2006, **29**, 120–129.
